# The Role of Information and Communications Technology Policies and Infrastructure in Curbing the Spread of the Novel Coronavirus: Cross-country Comparative Study

**DOI:** 10.2196/31066

**Published:** 2022-01-07

**Authors:** Nam Ji Eum, Seung Hyun Kim

**Affiliations:** 1 School of Business Yonsei University Seoul Republic of Korea

**Keywords:** health policy, telehealth, physical distancing, disease transmission, COVID-19

## Abstract

**Background:**

Despite worldwide efforts, control of COVID-19 transmission and its after effects is lagging. As seen from the cases of SARS-CoV-2 and influenza, worldwide crises associated with infections and their side effects are likely to recur in the future because of extensive international interactions. Consequently, there is an urgent need to identify the factors that can mitigate disease spread. We observed that the transmission speed and severity of consequences of COVID-19 varied substantially across countries, signaling the need for a country-level investigation.

**Objective:**

We aimed to investigate how distancing-enabling information and communications technology (ICT) infrastructure and medical ICT infrastructure, and related policies have affected the cumulative number of confirmed cases, fatality rate, and initial speed of transmission across different countries.

**Methods:**

We analyzed the determinants of COVID-19 transmission during the relatively early days of the pandemic by conducting regression analysis based on our data for country-level characteristics, including demographics, culture, ICT infrastructure, policies, economic status, and transmission of COVID-19. To gain further insights, we conducted a subsample analysis for countries with low population density.

**Results:**

Our full sample analysis showed that *implied telehealth policy*, which refers to the lack of a specific telehealth-related policy but presence of a general eHealth policy, was associated with lower fatality rates when controlled for cultural characteristics (*P*=.004). In particular, the fatality rate for countries with an implied telehealth policy was lower than that for others by 2.7%. Interestingly, *stated telehealth policy*, which refers to the existence of a specified telehealth policy, was found to not be associated with lower fatality rates (*P*=.30). Furthermore, countries with a government-run health website had 36% fewer confirmed cases than those without it, when controlled for cultural characteristics (*P*=.03). Our analysis further revealed that the interaction between implied telehealth policy and training ICT health was significant (*P*=.01), suggesting that implied telehealth policy may be more effective when in-service training on ICT is provided to health professionals. In addition, credit card ownership, as an enabler of convenient e-commerce transactions and distancing, showed a negative association with fatality rates in the full sample analysis (*P*=.04), but not in the subsample analysis (*P*=.76), highlighting that distancing-enabling ICT is more useful in densely populated countries.

**Conclusions:**

Our findings demonstrate important relationships between national traits and COVID-19 infections, suggesting guidelines for policymakers to minimize the negative consequences of pandemics. The findings suggest physicians’ autonomous use of medical ICT and strategic allocation of distancing-enabling ICT infrastructure in countries with high population density to maximize efficiency. This study also encourages further research to investigate the role of health policies in combatting COVID-19 and other pandemics.

## Introduction

First identified in December 2019, the novel COVID-19 outbreak has rapidly spread worldwide. As of September 23, 2021, the World Health Organization (WHO) reported that over 229.8 million people were infected worldwide, with over 4.7 million deaths caused by COVID-19 [1]. Furthermore, on December 19, 2020, a mutant of COVID-19, labeled B.1.1.7, was found, causing further havoc specifically in European countries. Consequently, the UK Prime Minister Boris Johnson imposed another lockdown that was even stricter than the previous lockdowns [2]. This kind of catastrophic pandemic is not unprecedented. From 1918 to 1919, the H1N1 influenza A virus emerged and went on to infect approximately 40% of the global population, with a mortality rate of more than 2% [3]. The H1N1 flu persists to this date, over 100 years since its first appearance, and has undergone significant genetic mutations. Virologists expect COVID-19 to follow a similar pattern [4,5]. As such, the prevalence of pandemics and genetic mutations is not a one-off phenomenon. Other outbreaks with disruptive social and economic consequences are probable [6], demanding research on how to control the spread of a virus in its early stages.

With this urgent need in mind, it is noteworthy that the infection and fatality rates as well as the speed of transmission have varied widely across countries. Countries, such as Israel, Singapore, South Korea, and Taiwan, are regarded as relatively successful in curbing transmission [7], whereas European countries and the United States experienced an explosive increase in the number of confirmed cases [1]. It is known that minimizing physical contact between individuals without disturbing their daily lives and improved medical practices are crucial in managing the spread of infectious diseases in general [8]. First, as a means of reducing physical contact and enabling social distancing, national information and communications technology (ICT) infrastructure, such as e-commerce and high speed internet connection, has played a key role in many countries [8,9]. Second, the possible importance of medical ICT policies and infrastructure has also been recognized. For example, effective use of telehealth practices has been credited with the successful management of other infectious diseases, such as severe acute respiratory syndrome (SARS) and Middle East respiratory syndrome (MERS), pointing to a possible role for telehealth in controlling pandemics [10]. In addition, other medical ICTs, such as computerized physician order entry (CPOE) and e-prescribing, have been acknowledged as drivers of health care improvements in quality and efficiency [11]. Third, countries differed significantly across other dimensions, such as implementation of early lockdown and conformity to government policies because of their cultural differences, which could have affected their success in social distancing.

Therefore, to gain broader insights, there is a need for country-level analysis of the national-level characteristics that mitigate the spread of COVID-19 through successful distancing and improved medical practices. Nevertheless, previous studies on the spread of COVID-19 tended to have narrower scopes, such as individuals, several cities, a single country, or a single continent, rather than a global focus [12-17]. Although there are some exceptions, those studies were still limited in the number of countries, possibly due to difficulty in data collection, for investigation [15,16]. For instance, the latest cross-country study on the effect of threat or coping appraisal on distancing compliance was conducted by comparing 5 countries only [18]. In addition, prior research also focused on how the demographic, cultural, or political factors of a country affected COVID-19 infection [19-22], but did not include ICT-related factors.

To address such a research gap, the primary objective of this study was to identify what national characteristics have a major impact on curtailing contagious diseases during the relatively early days of the pandemic. In particular, we focused on the role of distancing-enabling ICT infrastructure (DistancingICT) and medical ICT infrastructure and policy (MedicalICT) in containing COVID-19 infection, fatality rates, and transmission speed.

## Methods

### Data Collection

The main sources of country-level data used in this study included the United Nations, the World Bank, the WHO, Worldometers, Our World in Data (OWID), Ookla, Hofstede Insights, and Wikipedia. Among these, Worldometers is a reference site that provides real-time statistics on diverse topics. As a widely used source of research, media, and teaching, OWID is an online scientific publication institute that focuses on global issues such as poverty and disease. Ookla provides analyses of internet access performance metrics. Hofstede Insights provides culture scores of each country based on Hofstede’s cultural dimension theory [23], and is widely used in academic research. For example, to predict growth of COVID-19 confirmed cases across several countries, Hofstede dimensions are used to account for cultural factors [24]. Wikipedia is used only to determine which countries enforced national lockdowns in the early days of COVID-19. The accuracy of enforcement and the dates of enforcement were further confirmed through research in the media. All the data were collected between July and August 2020. This sampling strategy allows us to analyze the determinants of COVID-19 transmission during the early days of the pandemic.

**Figure 1:**
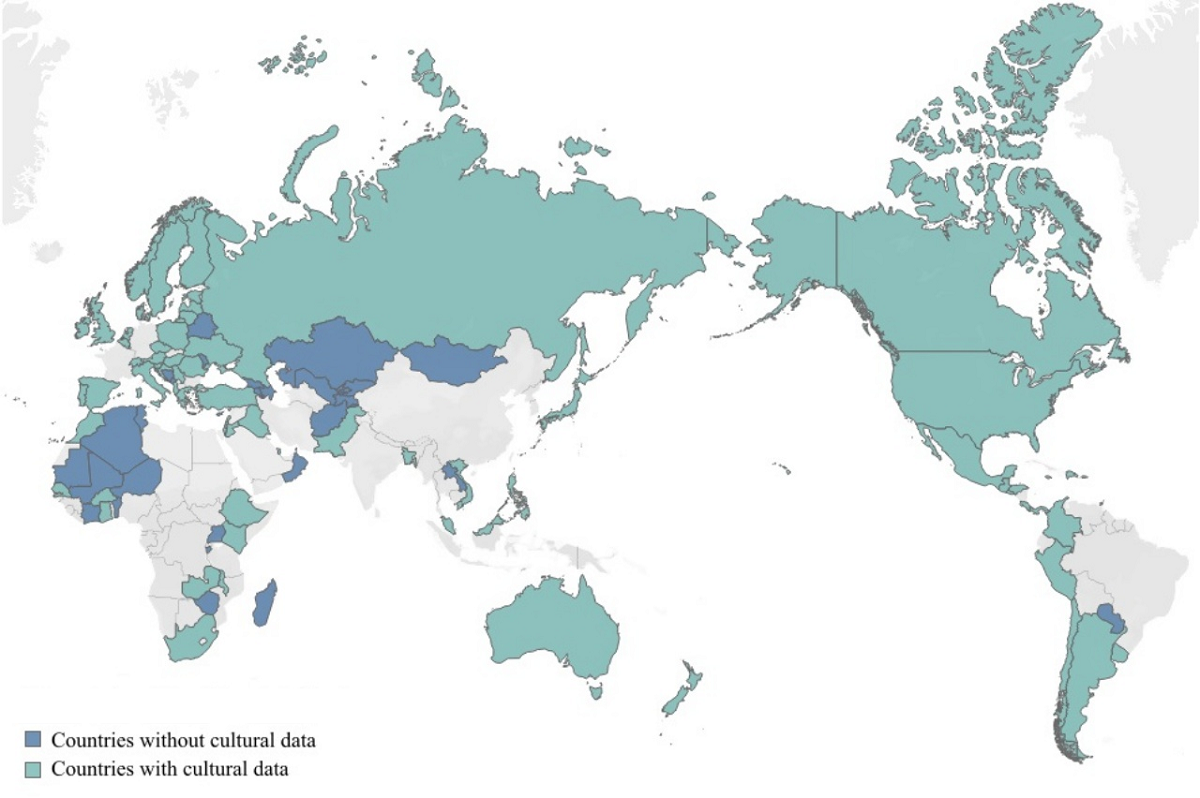
Map chart of the countries used in the analysis.

We limited our analysis to the countries that reported statistics related to the spread of COVID-19. For example, countries without accurate statistics on deaths as of July 28, 2020, were excluded. China, the country at the epicenter of COVID-19, was excluded because the patterns of disease spread and governmental control differ greatly from those in other countries. By matching the data for the social, economic, and demographic statuses of countries, as well as their physical distancing and health care–related ICT infrastructure, our final data set consisted of 98 countries. The countries included in our analysis are shown in Figure 1.

We focused on the following 3 dependent variables that represented the early state of the spread of COVID-19 in each country: (1) the cumulative number of infections, (2) the fatality rate, and (3) the number of days from initial infection to the 1000th infection. The first dependent variable represents the cumulative number of COVID-19 infections per country as of July 28, 2020. The second dependent variable is the fatality rate, which is the death toll over the cumulative number of infections. The third dependent variable is the transmission speed of COVID-19 in its initial stage. This is represented for each country by the number of days from the date of the first confirmed case to the date of the 1000th confirmed case. For the third dependent variable, countries with fewer than 1000 cases as of July 28, 2020, were excluded due to the difficulty of calculating the speed of transmission.

The 2 main categories of our main independent variables were DistancingICT and MedicalICT. For DistancingICT, we chose the following 2 variables based on findings of previous research: rate of credit card ownership and broadband internet speed. Research and Markets reported that during the COVID-19 pandemic, North America’s online sales surged, and credit cards were the top payment method for these online sales [25]. Previous studies further support the relationship between e-commerce and credit card usage. Meyll and Walter surveyed more than 25,000 US households and confirmed that individuals using mobile payments are likely to use credit cards [26]. Given this direct relationship between e-commerce and credit card usage, we identified credit cards as a major enabler of distancing as they serve as alternatives to offline shopping. Broadband internet speed also assists individuals to comply with stay-at-home orders during COVID-19 [27]. Next, the MedicalICT variables involved the availability of a national telehealth policy or strategy; availability of government-supported multilingual health internet websites that provide information; institutions with health care ICT training; and national electronic health records (EHRs). These variables represent MedicalICT because the variables relate to either reliable online sharing of medical information (government health internet sites and national EHRs) or the effective use of existing medical ICT technology (national telehealth policy and health care ICT training).

In particular, national telehealth policy is divided into the following 2 types: implied and stated. An *implied telehealth policy* means that a country does not have a specific national telehealth policy or strategy, and such a policy is referred to in the overall national eHealth policy. Australia, Finland, and the United States are examples of such countries. The United Kingdom and Norway, on the other hand, have separate telehealth policies. For instance, the Norwegian Ministry of Health commissioned the Norwegian Centre for Telemedicine to foster telehealth services, while assuring “the necessary actions to secure a successful dissemination of the services” [28]. As such, countries with a specific national telehealth policy or strategy, apart from a national eHealth strategy, are accounted for as “stated” telehealth policy in our model. Although the term telehealth and eHealth are at times used interchangeably, they differ in the purpose of use. While telehealth indicates usage of ICT to promote long-distance care, eHealth indicates usage of ICT for health in general. For example, in the United States, before COVID-19, telehealth was only for people who needed long-distance care due to limited access to nearby hospitals [29,30], while eHealth was widely applied to patients regardless of hospital accessibility. Accordingly, reimbursement on telehealth has not been prioritized or sufficiently instituted, as compared to that on general eHealth [30]. As such, telehealth, comparably, lacked clearly stated guidance before the pandemic.

Our control variables were selected based on the results of prior research that primarily focused on how the demographic, cultural, or political factors of a country affected COVID-19 infections [19-22]. The larger the scale of these countries’ economies, the greater their potential for economic activities, such as job hunting and international exchanges, that increase the opportunities for infections. Thus, we included gross domestic product (GDP) at purchasing power parity (PPP) and unemployment rate as controls for the economic statuses of countries. Moreover, because several studies have indicated that high temperatures and humidity may influence the infection rate of COVID-19, we included annual rainfall and temperature as controls [31]. Similarly, we added other controls, such as the proportion of senior citizens, early implementation of a national lockdown, and population density, to our model, along with 2 culture-related variables, individualism and uncertainty avoidance. Overall, we selected additional control variables that are identified as important determinants of the spread of contagious viruses in the literature. Detailed explanations of our main variables and additional control variables are summarized in Table 1.

**Table 1:** Variable descriptions.

Variable name		Description (by country)	Source	Year measured
**Dependent variable**				
	Total cases^a^	Number of individuals infected by COVID-19	Worldometers	2020
	Fatality rate^a^	Death rate against the number of confirmed cases (0-100)	Worldometers	2020
	Number of days^b^	Number of days elapsed from the first confirmed case to the 1000th confirmed case	Our World in Data	2020
**Control variable**				
	GDP^c^ PPP^d^	GDP by PPP in billions	World Bank	2019
	Unemployment rate	Unemployment rate (0-100)	World Bank	2019
	Population density	People per square km of land area	World Bank	2019
	Percent aged 60 or over	Percentage of people aged 60 or older	United Nations	2019
	Annual rainfall	Average annual rainfall in mm	World Bank	2019
	Annual temperature	Average annual temperature in °C	World Bank	2019
	Early lockdown	Implementation of a national lockdown within 1 month of the first confirmed case (dichotomous)	Wikipedia, Press	2020
	Individualism	Cultural dimension score for preference for a loosely knit social framework in which individuals are expected to take care of only themselves and their immediate families (0-100)	Hofstede^e^	2015
	Uncertainty avoidance	Cultural dimension score for degree to which the members of a society feel uncomfortable with uncertainty and ambiguity (0-100)	Hofstede	2015
**ICT^f^** **infrastructure enabling physical distancing**				
	Credit card ownership	The percentage of respondents who report having a credit card (aged 15+)	World Bank	2017
	Broadband speed	Broadband internet speed in Mbps	Ookla	2019
**Medical ICT infrastructure and policy**				
	Telehealth policy (stated)	Country with a stated telehealth policy or strategy (1: yes, 0: otherwise)	World Health Organization	2015
	Telehealth policy (implied)	Country with no specific telehealth policy or strategy but is referred in an overall eHealth policy or strategy (1: yes, 0: otherwise)	World Health Organization	2015
	Government health websites	Government-supported health internet sites providing information (1: available, 0: not available)	World Health Organization	2015
	Training ICT health	Institutions offering in-service training to health professionals on ICT for health (1: available, 0: not available)	World Health Organization	2015
	National EHR^g^	Country with a national EHR (1: available, 0: not available)	World Health Organization	2015

^a^Data collected as of July 28, 2020.

^b^Data collected as of August 9, 2020.

^c^GDP: gross domestic product.

^d^PPP: purchasing power parity.

^e^Definitions for Hofstede variables were obtained online [32].

^f^ICT: information and communications technology.

^g^EHR: electronic health record.

### Empirical Analysis

For each dependent variable, we specified our models as follows:

*log* (*Total Cases_i_*) = *α_1_* + *β_11_Control_i_* + *β_12_DistancingICT_i_* + *β_13_MedicalICT_i_* + *ε_1i_*
** (1)**

*Fatality Rate_i_* = *α_2_* + *β_21_Control_i_* + *β_22_DistancingICT_i_* + *β_23_MedicalICT_i_* + *ε_2i_*
** (2)**

*log (Number of Daysi) = α_3_ + β_31_Control_i_ + β_32_DistancingICT_i_ + β_33_MedicalICT_i_ + ε_3i_*
** (3)**

where *i* denotes an individual country.

For simplicity, an ordinary least squares estimator was used to estimate the coefficients. The variables of main interest were DistancingICT and MedicalICT. The positive coefficient values of DistancingICT and MedicalICT in equations 1 and 2 demonstrate that the variables increased the total number of confirmed cases and the fatality rate. In contrast, the positive coefficients of DistancingICT and MedicalICT in equation 3 represent a slower transmission speed. In all 3 models, we used the same set of control variables, including GDP PPP, unemployment rate, population density, elderly population ratio, annual rainfall, annual temperature, and early lockdown. For normality, we log transformed all the variables, including total cases and number of days, that displayed skewed distributions and were nonnegative.

For each dependent variable, our baseline model included all the main independent variables and controls, but without the 2 culture-related variables of individualism and uncertainty avoidance. In the second model, we added these culture-related variables to the baseline model. We performed this separate estimation because the content for the culture-related variables was not available for all 98 countries. Thus, adding them to the model reduced the sample size from 98 to 69. In the third model, we added several interaction terms to check for possible interaction effects between MedicalICT variables. To ensure that independent variables in the analysis were not correlated, we calculated variance inflation factor (VIF). All the independent variables had VIF values less than 10, which indicates no multicollinearity violations [33]. Lastly, robust standard errors were used to address any possible heteroskedastic error.

We also conducted a subsample analysis in which we removed countries with high population density. A prior study showed positive correlation between population density and COVID-19 infection [14]. Residents of densely populated countries inevitably have interactions and contacts with more people offline, whereas loosely populated countries can reduce such possibilities when the infrastructure is built up. Moreover, less populated countries may not have sufficient localized medical services, thus requiring more medical ICT infrastructure than other countries. The needs and utilization of ICT would significantly vary by population density as well. Thus, to determine if our results might be biased by the inclusion of countries with higher population density, we conducted an additional analysis with only countries with lower population density.

## Results

### Main Analysis

The descriptive statistics for the variables used in this analysis and their correlations are presented in Table 2 and Multimedia Appendix 1, respectively.

**Table 2:** Summary statistics.

Variable name		Mean (SD) value	Minimum value	Maximum value
**Dependent variable**				
	Total cases, n (98 countries)	113,021.2 (464,974.3)	20	4,498,343
	Fatality rate, % (95 countries)	3.32 (3.18)	0.05	15.26
	Number of days, n (91 countries)	51.02 (29.30)	11	139
**Control variables**				
	GDP^a^ PPP^b^, US $ billions (98 countries)	734.76 (2,290.02)	9.03	21,427.70
	Unemployment rate, % (98 countries)	6.55 (4.68)	0.09	28.18
	Population density, persons/km^2^ (98 countries)	256.50 (919.32)	2.11	8737.02
	Percentage aged 60 or older, % (98 countries)	15.32 (8.85)	3.19	34.02
	Annual rainfall, mm (98 countries)	78.85 (54.56)	1.53	244.87
	Annual temperature, °C (98 countries)	16.74 (8.57)	−4.97	29.29
	Early lockdown, dichotomous (98 countries)	0.36 (0.48)	0	1
	Individualism, numeric score (68 countries)	42.85 (23.57)	6	91
	Uncertainty avoidance, numeric score (68 countries)	66.97 (22.67)	8	100
**Distancing-enabling ICT^c^** **infrastructure**				
	Credit card ownership rate, % (98 countries)	21.23 (22.25)	0	83
	Broadband speed, Mbps (98 countries)	45.47 (36.40)	4.18	191.93
**Medical ICT infrastructure**				
	Telehealth policy (stated), dichotomous (98 countries)	0.22 (0.42)	0	1
	Telehealth policy (implied), dichotomous (98 countries)	0.37 (0.49)	0	1
	Government health websites, dichotomous (98 countries)	0.61 (0.49)	0	1
	Training ICT health, dichotomous (98 countries)	0.82 (0.39)	0	1
	National EHR^d^, dichotomous (98 countries)	0.47 (0.50)	0	1

^a^GDP: gross domestic product.

^b^PPP: purchasing power parity.

^c^ICT: information and communications technology.

^d^EHR: electronic health record.

The results for regressions with robust standard error are shown in Tables 3-5. For each dependent variable (number of confirmed cases [Table 3], fatality rate [Table 4], and transmission speed [Table 5]), model 1 was the baseline model, while model 2 added culture-related variables as controls. Model 3 included all the control variables as well as the interaction effects between distancing ICT and medical ICT variables (*DistancingICT_i_* × *MedicalICT_i_*). As mentioned above, in the case of *Number of Days_i_*, countries with no reported cases of the 1000th infection as of July 28, 2020, were excluded from the analysis. As for the goodness of fit, *R^2^* for *Total Cases_i_* was higher than that for *Fatality Rate_i_* and *Number of Days_i_*, indicating that the national characteristic variable used in the analysis explains the cumulative number of infected better than the 2 other dependent variables. For *Total Cases_i_*, the *R^2^* values for the 3 models were 61.5%, 73.3%, and 75.4%, respectively. Considering that the *R^2^* averages of *Fatality Rate_i_* and *Number of Days_i_* were 44.5% and 37.0%, respectively, the overall explanatory power of the models for *Total Cases_i_* exceeded that of the 2 others. Therefore, national characteristics account for a significant portion of the differences in the cumulative number of confirmed cases by country.

**Table 3:** Main regression results for the full sample with the dependent variable log (total cases).

Variable		Model 1^a^ (baseline model), regression coefficient (SE)	*P* value	Model 2^b^ (including culture), regression coefficient (SE)	*P* value	Model 3^c^ (interaction effects), regression coefficient (SE)	*P* value
**General variable**							
	log (GDP^d^ PPP^e^)	1.136 (0.116)	<.001	1.124 (0.122)	<.001	1.104 (0.133)	<.001
	Unemployment rate	0.033 (0.016)	.04	0.015 (0.018)	.40	0.019 (0.019)	.32
	log (population density)	0.250 (0.129)	.06	0.278 (0.150)	.07	0.359 (0.162)	.03
	Percentage aged 60 years or older	−0.027 (0.014)	.06	−0.054 (0.019)	.006	−0.055 (0.020)	.007
	log (annual rainfall)	−0.253 (0.169)	.12	−0.023 (0.246)	.93	−0.115 (0.258)	.66
	Annual temperature	−0.021 (0.012)	.07	−0.030 (0.015)	.06	−0.035 (0.016)	.03
	Early lockdown	−0.018 (0.151)	.91	0.04 (0.158)	.80	−0.06 (0.179)	.74
	Individualism	N/A^f^	N/A	0.005 (0.006)	.42	0.005 (0.006)	.37
	Uncertainty avoidance	N/A	N/A	0.007 (0.004)	.08	0.006 (0.004)	.15
**Distancing-enabling ICT^g^** **infrastructure**							
	Credit card ownership rate	−0.002 (0.005)	.64	0.0002 (0.005)	.97	−0.0002 (0.005)	.97
	log (broadband speed)	0.068 (0.316)	.83	0.156 (0.335)	.64	0.104 (0.364)	.78
**Medical ICT infrastructure**							
	Telehealth policy (stated)	0.055 (0.179)	.76	0.192 (0.191)	.32	0.861 (0.648)	.19
	Telehealth policy (implied)	−0.003 (0.152)	.98	−0.098 (0.166)	.56	−0.13 (0.433)	.77
	Government health websites	−0.221 (0.161)	.17	−0.440 (0.193)	.03	−0.675 (0.278)	.02
	Training ICT health	0.174 (0.183)	.35	0.096 (0.205)	.64	0.276 (0.311)	.38
	National EHR^h^	0.179 (0.137)	.20	0.094 (0.144)	.52	0.206 (0.231)	.38
**Interaction**							
	Telehealth policy (stated)×government health websites	N/A	N/A	N/A	N/A	−0.049 (0.443)	.91
	Telehealth policy (implied)×government health websites	N/A	N/A	N/A	N/A	0.527 (0.351)	.14
	Telehealth policy (stated)×training ICT health	N/A	N/A	N/A	N/A	−0.567 (0.758)	.46
	Telehealth policy (implied)×training ICT health	N/A	N/A	N/A	N/A	−0.241 (0.417)	.57
	Telehealth policy (stated)×national EHR	N/A	N/A	N/A	N/A	−0.117 (0.442)	.79
	Telehealth policy (implied)×national EHR	N/A	N/A	N/A	N/A	−0.25 (0.442)	.52
Constant		−8.285 (1.347)	<.001	−8.577 (1.579)	<.001	−8.140 (1.745)	<.001

^a^Model 1: 98 observations; R^2^=0.615; adjusted R^2^=0.55.

^b^Model 2: 69 observations; R^2^=0.733; adjusted R^2^=0.65.

^c^Model 3: 69 observations; R^2^=0.754; adjusted R^2^=0.64.

^d^GDP: gross domestic product.

^e^PPP: purchasing power parity.

^f^N/A: not applicable.

^g^ICT: information and communications technology.

^h^EHR: electronic health record.

**Table 4:** Main regression results for the full sample with the dependent variable fatality rate.

Variable		Model 1^a^ (baseline model), regression coefficient (SE)	*P* value	Model 2^b^ (including culture), regression coefficient (SE)	*P* value	Model 3^c^ (interaction effects), regression coefficient (SE)	*P* value
**General variable**							
	log (GDP^d^ PPP^e^)	1.635 (0.571)	.005	1.573 (0.670)	.02	1.993 (0.711)	.007
	Unemployment rate	−0.042 (0.077)	.59	−0.019 (0.095)	.84	0.043 (0.099)	.67
	log (population density)	0.036 (0.623)	.95	1.579 (0.805)	.06	1.962 (0.843)	.03
	Percentage aged 60 or older	0.143 (0.07)	.04	0.045 (0.101)	.66	0.025 (0.103)	.81
	log (annual rainfall)	0.198 (0.835)	.81	2.373 (1.325)	.08	2.224 (1.346)	.11
	Annual temperature	−0.011 (0.058)	.85	−0.033 (0.082)	.69	−0.041 (0.082)	.62
	Early lockdown	−0.617 (0.737)	.41	−1.000 (0.860)	.25	−0.535 (0.931)	.57
	Individualism	N/A^f^	N/A	0.138 (0.031)	<.001	0.141 (0.031)	<.001
	Uncertainty avoidance	N/A	N/A	0.034 (0.022)	.13	0.036 (0.022)	.11
**Distancing-enabling ICT^g^** **infrastructure**							
	Credit card ownership rate	0.0002 (0.023)	.99	−0.057 (0.027)	.04	−0.074 (0.028)	.01
	log (broadband speed)	−1.287 (1.513)	.40	−1.199 (1.799)	.51	−0.015 (1.893)	.99
**Medical ICT infrastructure**							
	Telehealth policy (stated)	−0.715 (0.888)	.42	−1.093 (1.042)	.30	−1.03 (3.367)	.76
	Telehealth policy (implied)	−1.176 (0.743)	.12	−2.684 (0.903)	.004	−6.231 (2.242)	.008
	Government health websites	−0.053 (0.800)	.95	−1.721 (1.058)	.11	−2.985 (1.473)	.05
	Training ICT health	−0.37 (0.904)	.68	−0.091 (1.100)	.93	0.029 (1.611)	.99
	National EHR^h^	−0.508 (0.655)	.44	0.888 (0.780)	.26	−0.206 (1.208)	.87
**Interaction**							
	Telehealth policy (stated)×government health websites	N/A	N/A	N/A	N/A	2.896 (2.315)	.22
	Telehealth policy (implied)×government health websites	N/A	N/A	N/A	N/A	1.7 (1.832)	.36
	Telehealth policy (stated)×training ICT health	N/A	N/A	N/A	N/A	−0.595 (3.934)	.88
	Telehealth policy (implied)×training ICT health	N/A	N/A	N/A	N/A	1.654 (2.162)	.45
	Telehealth policy (stated)×national EHR	N/A	N/A	N/A	N/A	−1.256 (2.293)	.59
	Telehealth policy (implied)×national EHR	N/A	N/A	N/A	N/A	3.419 (2.009)	.10
Constant		−13.999 (6.791)	.04	−24.255 (8.600)	.007	−30.459 (9.241)	.002

^a^Model 1: 95 observations; R^2^=0.271; adjusted R^2^=0.143.

^b^Model 2: 68 observations; R^2^=0.496; adjusted R^2^=0.338.

^c^Model 3: 68 observations; R^2^=0.569; adjusted R^2^=0.358.

^d^GDP: gross domestic product.

^e^PPP: purchasing power parity.

^f^N/A: not applicable.

^g^ICT: information and communications technology.

^h^EHR: electronic health record.

**Table 5:** Main regression results for the full sample with the dependent variable log (number of days).

Variable		Model 1^a^ (baseline model), regression coefficient (SE)	*P* value	Model 2^b^ (including culture), regression coefficient (SE)	*P* value	Model 3^c^ (interaction effects), regression coefficient (SE)	*P* value
**General variable**							
	log (GDP^d^ PPP^e^)	−0.130 (0.044)	.005	−0.08 (0.055)	.15	−0.061 (0.058)	.29
	Unemployment rate	−0.001 (0.006)	.84	−0.001 (0.007)	.87	0.004 (0.008)	.61
	log (population density)	−0.068 (0.047)	.16	−0.089 (0.064)	.17	−0.079 (0.066)	.24
	Percentage aged 60 or older	0.005 (0.005)	.30	0.01 (0.008)	.23	0.003 (0.008)	.71
	log (annual rainfall)	0.032 (0.063)	.62	−0.05 (0.104)	.63	−0.057 (0.105)	.59
	Annual temperature	0.010 (0.005)	.03	0.008 (0.007)	.21	0.006 (0.007)	.36
	Early lockdown	−0.096 (0.055)	.09	−0.118 (0.069)	.09	−0.118 (0.075)	.13
	Individualism	N/A^f^	N/A	−0.002 (0.002)	.46	−0.002 (0.002)	.46
	Uncertainty avoidance	N/A	N/A	−0.003 (0.002)	.17	−0.002 (0.002)	.26
**Distancing-enabling ICT^g^** **infrastructure**							
	Credit card ownership rate	−0.0003 (0.002)	.88	−0.001 (0.002)	.56	0.0004 (0.002)	.88
	log (broadband speed)	−0.125 (0.113)	.27	−0.208 (0.140)	.14	−0.260 (0.146)	.08
**Medical ICT infrastructure**							
	Telehealth policy (stated)	0.002 (0.066)	.98	0.009 (0.081)	.91	−0.328 (0.260)	.21
	Telehealth policy (implied)	0.001 (0.055)	.99	0.042 (0.071)	.56	−0.357 (0.183)	.06
	Government health websites	0.017 (0.061)	.78	0.047 (0.085)	.59	0.067 (0.117)	.57
	Training ICT health	0.055 (0.070)	.44	0.042 (0.091)	.65	−0.203 (0.128)	.12
	National EHR^h^	−0.029 (0.049)	.55	0.018 (0.062)	.77	0.038 (0.096)	.70
**Interaction**							
	Telehealth policy (stated)×government health websites	N/A	N/A	N/A	N/A	−0.015 (0.183)	.94
	Telehealth policy (implied)×government health websites	N/A	N/A	N/A	N/A	0.02 (0.144)	.89
	Telehealth policy (stated)×training ICT health	N/A	N/A	N/A	N/A	0.415 (0.304)	.18
	Telehealth policy (implied)×training ICT health	N/A	N/A	N/A	N/A	0.506 (0.188)	.01
	Telehealth policy (stated)×national EHR	N/A	N/A	N/A	N/A	−0.013 (0.178)	.94
	Telehealth policy (implied)×national EHR	N/A	N/A	N/A	N/A	−0.107 (0.159)	.50
Constant		3.131 (0.520)	<.001	3.078 (0.690)	<.001	3.163 (0.723)	<.001

^a^Model 1: 91 observations; R^2^=0.341; adjusted R^2^=0.219.

^b^Model 2: 65 observations; R^2^=0.329; adjusted R^2^=0.105.

^c^Model 3: 65 observations; R^2^=0.440; adjusted R^2^=0.146.

^d^GDP: gross domestic product.

^e^PPP: purchasing power parity.

^f^N/A: not applicable.

^g^ICT: information and communications technology.

^h^EHR: electronic health record.

Our results suggest that medical ICT policy, rather than the ICT infrastructure itself, is negatively associated with the fatality rate. For example, the coefficient for implied telehealth policy was −2.684 and significant (*P*=.004) (Table 4), that is, the fatality rate for countries with an implied telehealth policy was lower than that for others by 2.7 percentage points. Moreover, the coefficient for the rate of credit card ownership was −0.057 and significant (*P*=.04) (Table 4), suggesting that credit card usage could have lessened the fatality rate. However, broadband internet speed was not associated with any of the 3 measures of transmission of COVID-19. Lastly, the presence of a government-run health website showed a negative and significant relationship with the total number of confirmed cases (β=−0.440; *P*=.03) (Table 3). This implies that countries with government-run health websites had 36% fewer confirmed cases than those without it.

The effects of DistancingICT and MedicalICT on the transmission speed of COVID-19 were not statistically significant. For the interaction terms, although most coefficients were insignificant, the interaction between implied telehealth policy and training ICT health was significant (β=0.506; *P*=.01) (Table 5). Therefore, an implied telehealth policy may be more effective when in-service training on ICT is provided to health professionals (β=−0.357+0.506=0.149).

Despite this not being the main focus of the study, it would be meaningful to examine the effects of other control variables in light of the lack of country-level empirical studies on the spread of COVID-19. Interestingly, early lockdowns, contrary to expectations, were statistically uncorrelated with the total number of infections and the fatality rate. Moreover, the coefficients for GDP with the total number of confirmed cases and the fatality rate were 1.136 (*P*<.001) and 1.635 (*P*=.005), respectively (Table 4). Population density was positively associated with the total number of cases and the fatality rate, but temperature was negatively associated with the total number of infections. Lastly, the ratio of the elderly population and the total number of infections showed a negative relationship (β=−0.027; *P*=.06) (Table 3).

As for the cultural dimensions of COVID-19, our results suggested no significant relationship between the fatality rate and uncertainty avoidance, 1 of the 2 cultural dimensions from Hofstede Insights. However, individuals’ tendency to care only for themselves and their immediate family, as represented by individualism, showed a positive relationship with the fatality rate (β=0.138; *P*<.001) (Table 4).

### Additional Analysis

Although it has not been long since the COVID-19 outbreak and the transmission mechanism of the virus has not yet been clarified, it is apparent that human-to-human interaction increases the risk of infection [14,34]. Moreover, prior research has found a positive association between dense populations and infection rates [14], possibly because high density enhances the probability of an individual’s exposure to the virus. However, people in such areas could be more aware of the risk, consequently taking precautions or complying with government regulations to avoid an epidemic. Moreover, highly concentrated urbanization is more likely to offer entrenched contact-free systems (eg, delivery and retail kiosks) than less populated regions, thus stagnating or reducing the spread of the virus. As such, the effect of DistancingICT or MedicalICT may vary substantially between countries with high and low population densities. Therefore, we conducted a further regression analysis on countries outside of the top 30% in population density in our sample. The results of the further analysis are shown in Tables S1-3 in Multimedia Appendix 2.

The key results are not remarkably different. Telehealth policy, rather than technology itself, may promote efficient management of COVID-19’s aftermath regardless of population density. Unlike other national traits, the presence of telehealth policy (stated and implied) showed a negative association with the fatality rate when we omitted countries in the top 30% of population density from our sample. These consistent results highlight the importance of telehealth policy development in infection containment. However, it is noteworthy that credit card ownership was no longer significant. It is conceivable that countries with lower population density also have lesser rates of offline physical interaction, thus lowering the need for credit cards and online shopping.

## Discussion

### Principal Findings

In this study, we conducted exploratory research on the role of national characteristics, especially in regard to ICT and medical ICT infrastructure and concomitant policies that enable physical distancing, in the cumulative number of confirmed cases, fatality rates, and initial transmission speed of COVID-19. The findings suggested that medical ICT policies, especially when in-service training on ICT is provided, could potentially reduce the fatality rate. Government health websites were negatively associated with the total number of confirmed cases. Moreover, possession of a credit card was observed to decrease the fatality rate.

### Discussion of the Findings

The analysis results countered general intuition that ICT infrastructure should play a crucial role in slowing COVID-19 transmission. Overall, we found that the relationship between ICT infrastructure and COVID-19 infection or its consequences was less than expected. Nevertheless, there are some important findings to highlight.

First, the presence of a telehealth policy manifested a negative correlation with the fatality rate. Surprisingly, only implied telehealth policy, but not stated telehealth policy, showed a statistically significant correlation with the fatality rate. This raises the possibility that an implied telehealth policy may be more effective than a stated one because setting specific guidelines as in a stated policy sets boundaries that hinder clinicians’ flexible decision-making or system utilization in a crisis [35]. The result remained consistent even when countries with high population concentrations were excluded. Moreover, it is important to note that the telehealth policy becomes more relevant when in-service training on ICT is provided. Such an interaction result is aligned with WHO Digital Health Guidelines that state “Extensive training on the technology and operating the device should be done before introducing the system for use directly with clients” [36]. It is also notable that government health websites had a negative and significant relationship with the total number of confirmed cases. Consistent with preceding studies [37,38], it advocates governmental online communication in case of disease outbreaks to facilitate efficient interconnection between specialists from various fields. Otherwise, prompt and precise communication attempts by the government could enhance information transparency and build trust among the public, increasing the likelihood of public compliance with suggested guidelines including vaccine acceptance [39].

Second, our findings suggest that possession of a credit card, a widely used payment method for e-commerce [25,26], is related to a lower fatality rate. The results varied when countries with higher population density were omitted; this could happen because of either uneven development of contact-free systems in areas of lower density or prevention of crowding through widespread interventions such as contact tracing [40]. Nonetheless, such a finding denotes how credit card ownership facilitates distancing compliance via online commerce. Alternatively, credit cards could have advanced financial inclusion and cushioned the financial burden even in challenging times. For instance, cardholders, especially those with a low income, have benefited from financial assistance programs, including deferred payments, waived late fees, and even skipped payments, from credit card issuers [41]. With financial assistance, perceived burden would have been decreased, fostering adherence to suggested guidelines [42].

Third, we had interesting findings from the control variables on the spread of COVID-19 and its aftermath. Early lockdown enforcement displayed no relationship with COVID-19 transmission, although policymakers intuitively assumed that compulsory restriction of contacts would be helpful in diminishing the total number of infections, fatality rate, and speed of transmission. Such intuitions are inferred from their actions to tighten stay-at-home restrictions [27]. Our result is consistent with the outcome of a preceding analysis that stated infection control was apparently effective before the mandate, and thus, voluntary social behavior, rather than legal enforcement, is more crucial in combating a pandemic [43,44].

Stronger individualistic culture exhibited a higher fatality rate, whereas uncertainty avoidance showed no association with the fatality rate. Related to individualism, a recent study clarified the role of individualism-collectivism on the perceived risk of COVID-19 and sense of responsibility [45]. In essence, individuals with strong collectivistic orientation perceived greater fear because of their higher physical and social interconnection with others [45]. Moreover, collectivism-oriented people, due to their strong sense of integrity and responsibility within the society, were willing to follow containment guidelines, whereas individualism-oriented people were not willing to follow guidelines [46]. This finding is in line with preceding research that identified a relationship between pathogen risk and societal individualism [47]. Societal collectivism, which is more prevalent in Eastern cultures, did “[serve] as a natural guard against disease transmission” [48].

A previous study also revealed that countries with relatively high uncertainty avoidance were less likely to engage in public gathering, potentially decreasing the number of infections and the fatality rate [21]. However, in this study, such an effect was not observed. The discrepancy could stem from different data collection periods. Huynh conducted an analysis at the initial stage of COVID-19, from February 16, 2020, to March 29, 2020 [21], but we used COVID-19 transmission data until July 28, 2020. It is challenging to refrain from public gatherings for several months; the impact of uncertainty avoidance would be weakened eventually.

The analysis result indicates that GDP is positively related to the number of total cases and the fatality rate. It is plausible that economically active countries, as represented by higher GDP, involve more interactions between individuals, causing an inevitable increase in the number of infections. Alternatively, in larger economies, the number of confirmed cases may reflect better testing because these countries have the economic capacity to conduct more tests. Lastly, a higher proportion of the elderly population was correlated with a fewer number of infections in total. The perceived risk of infection among elders might have influenced stay-at-home compliance, thus reducing physical contact and infections.

### Implications for Research and Practice

The results of this study have several implications. Theoretically, the findings contribute to the effect of ICT infrastructure and policy in epidemics. Prior studies have examined ICT adoption intention of health care workers or how ICT use improves public health or physical wellness in general [49-52]. Moreover, past research on ICT and health have paid attention to how ICT mitigates various health-related challenges by providing access to health-related information and fostering communication between patients and physicians [53-55]. For example, previous research found that telephone usage for health care lowered depression rates [53] and increased immunization rates [54]. However, they rarely showed interest for ICT use in the context of epidemics, possibly due to its unlikelihood. Similarly, previous research on ICT policy in health care mainly focused on the “limitations concerning design and implementation of policies” of public health improvement. Considering that ICT, by its nature, enables faster communication to the public, it is surprising that the effect of ICT on epidemics, which are widespread and abrupt, was not investigated sufficiently. In this study, we have addressed this void by examining the relationship of ICT policy with total cases, fatality rate, and transmission speed under the pandemic circumstance. Therefore, by expanding the scope of the role of ICT on people’s health, this study contributes to the literature on ICT and health-related challenges.

Practically, this study advocates autonomous use of medical ICT, rather than playing it by the book. Contrary to the assumption that detailed and rigorous policy statements limit or prevent a wide range of health threats, such as smoking habits and cardiovascular diseases [56,57], the findings indicate that stated medical ICT policy is in fact less likely to taper the fatality rate than implied medical ICT policy. It is plausible that relatively less restrictions are helpful for better medical services because for jobs with high variety tasks, such as medical practice, increased autonomy boosts the job performance of workers [58]. This finding is relevant in the broader context of the digital health field and provides significant empirical insights that could improve the outcome of long-distance medical care and guide future clinical decision support system (CDSS)-related strategies. This finding is aligned with WHO guidelines, which suggest that “health workers may deviate from the recommendations” of a CDSS based on physicians’ own rationale [36], although an algorithm-based CDSS is conventionally perceived as competent. As such, policymakers need to consider the independent and flexible decision-making of physicians in the context of medical ICT usage.

Regarding distancing-enabling infrastructure, this study showed that the government should prioritize providing ICT infrastructure that enables physical distancing in densely populated areas. As the budget for ICT infrastructure is limited, the government should strategically allocate funds to achieve the greatest benefit. Especially, strategic budgeting is vital in developing countries where tax revenue is relatively insufficient. Because our findings show that differences in population density yield different outcomes of ICT implementation, governors can consider investing in populous areas first, in order to maximize the benefit with limited resources.

### Conclusions

Despite our findings on the relationship between national characteristics and disease dispersion, our study is not without limitations. Although we included most established countries, we were not able to include all countries in our analysis. Consequently, the sample size for regression was small. In addition, the data for medical ICT infrastructure and the rate of credit card ownership were not up-to-date. Therefore, their impacts during the observation period may not have been accurately estimated. However, it is important to note that ICT policy and infrastructure have a delayed “lag” effect on country-level outcomes because people need to adopt, trust, and alter their behaviors in line with new technologies and policies [59]. For instance, a recent study on the role of ICT in women’s health outcomes showed that the maternal fatality rate was lowered while modern medical care seeking behavior increased after kiosks were implemented and used for some years [59]. In addition, although we included broadband speed in the model, broadband coverage may also play a distinct role. For instance, high broadband speed offers fast communication online, advocating real-time information sharing in dire situations such as COVID-19 [9]. On the other hand, broadband coverage enables seamless internet connectivity with personal devices; when individuals get out of the service range at some point, they would not get broadband access. While lack of decent broadband coverage indicates inability to use the internet, lack of decent broadband speed denotes unattainability of prompt communication with others. Nevertheless, the correlation between broadband coverage and speed was high (correlation=0.79). Accordingly, only one of them was used for our analysis to avoid a multicollinearity problem. Moreover, there are other potential confounders, such as mask adherence, that were not included in our study. However, we believe that our culture-related variables, such as individualism and uncertainty avoidance, may account for such confounders [60]. Lastly, because the COVID-19 pandemic has not ended, the long-term effect of ICT infrastructure and ICT policies could not be examined.

By conducting an analysis at the country level, we ensured the generalizability of our work and developed tentative guidelines to control the spread of infectious diseases. We have especially emphasized the importance of medical telehealth policies that contribute to reduce the consequences of COVID-19. By collecting updated COVID-19 data, future research can clarify the long-term effects of the aforementioned national traits. We hope that this study will broaden the scope of research on the impact of ICT infrastructure and policies, and give guidance for better policy-making in the health care domain.

## Appendix

Multimedia Appendix 1 Pairwise correlations (N=98).

Multimedia Appendix 2 Regression results for the sample with less population density (lower 70%).

## Abbreviations

CDSS: clinical decision support system
DistancingICT: distancing-enabling ICT infrastructure
EHR: electronic health record
GDP: gross domestic product
ICT: information and communications technology
MedicalICT: medical ICT infrastructure and policy
OWID: Our World in Data
PPP: purchasing power parity
VIF: variance inflation factor
WHO: World Health Organization
