# Supplementary material for: The Role of Information and Communications Technology Policies and Infrastructure in Curbing the Spread of the Novel Coronavirus: Cross-country Comparative Study
Source: JMIR Public Health Surveill. 2022 Jan 7;8(1):e31066. doi: 10.2196/31066 (PMC8745697; doi:10.2196/31066)
Supplement: Multimedia Appendix 1 [file publichealth_v8i1e31066_app1.docx]

**Multimedia Appendix 1.** Pairwise correlations (N=98).

|  | (1) | (2) | (3) | (4) | (5) | (6) | (7) | (8) | (9) | (10) | (11) | (12) | (13) | (14) | (15) | (16) | (17) | (18) |
| --- | --- | --- | --- | --- | --- | --- | --- | --- | --- | --- | --- | --- | --- | --- | --- | --- | --- | --- |
|  |  |  |  |  |  |  |  |  |  |  |  |  |  |  |  |  |  |  |
| **Dependent Variables** | | | | | | | | | | | | | | | | | | |
| (1) Total Cases |  |  |  |  |  |  |  |  |  |  |  |  |  |  |  |  |  |  |
| (2) Fatality Rate | 0.06 |  |  |  |  |  |  |  |  |  |  |  |  |  |  |  |  |  |
| (3) Number of Days | -0.08 | -0.23 |  |  |  |  |  |  |  |  |  |  |  |  |  |  |  |  |
| **Control Variables** | | | | | | | | | | | | | | | | | | |
| (4) GDP PPP | 0.95 | 0.15 | -0.08 |  |  |  |  |  |  |  |  |  |  |  |  |  |  |  |
| (5) Unemployment Rate | -0.02 | -0.03 | 0.03 | -0.08 |  |  |  |  |  |  |  |  |  |  |  |  |  |  |
| (6) Population Density | -0.03 | -0.12 | 0.05 | -0.02 | -0.13 |  |  |  |  |  |  |  |  |  |  |  |  |  |
| (7) Percent Ages 60 or Older | 0.09 | 0.41 | -0.30 | 0.20 | 0.10 | 0.03 |  |  |  |  |  |  |  |  |  |  |  |  |
| (8) Annual Rainfall | 0.07 | -0.05 | -0.09 | 0.06 | -0.04 | 0.08 | -0.11 |  |  |  |  |  |  |  |  |  |  |  |
| (9) Annual Temperature | 0.01 | -0.38 | 0.07 | -0.05 | -0.04 | -0.01 | -0.36 | 0.25 |  |  |  |  |  |  |  |  |  |  |
| (10) Early Lockdown | -0.09 | 0.00 | 0.04 | -0.12 | 0.09 | -0.08 | -0.07 | 0.03 | 0.01 |  |  |  |  |  |  |  |  |  |
| (11) Individualism | -0.04 | 0.59 | -0.22 | 0.06 | -0.05 | -0.08 | 0.48 | -0.38 | -0.65 | -0.25 |  |  |  |  |  |  |  |  |
| (12) Uncertainty Avoidance | 0.16 | -0.04 | 0.09 | 0.17 | 0.03 | 0.11 | -0.04 | -0.21 | -0.11 | 0.31 | -0.25 |  |  |  |  |  |  |  |
| **Distancing Enabling ICT Infrastructure** | | | | | | | | | | | | | | | | | | |
| (13) Credit Card Ownership Rate | 0.10 | 0.37 | -0.16 | 0.20 | 0.02 | 0.09 | 0.43 | 0.03 | -0.50 | -0.21 | 0.68 | -0.09 |  |  |  |  |  |  |
| (14) Broadband Speed | 0.20 | 0.29 | -0.31 | 0.28 | -0.22 | 0.40 | 0.66 | -0.15 | -0.30 | -0.20 | 0.36 | 0.02 | 0.38 |  |  |  |  |  |
| **Medical ICT Infrastructure** | | | | | | | | | | | | | | | | | | |
| (15) Telehealth Policy (Stated) | 0.19 | 0.04 | 0.01 | 0.19 | 0.01 | -0.08 | 0.09 | 0.14 | -0.06 | 0.10 | 0.01 | 0.02 | 0.16 | 0.05 |  |  |  |  |
| (16) Telehealth Policy (Implied) | -0.09 | 0.07 | -0.05 | -0.02 | -0.14 | -0.07 | -0.03 | -0.12 | -0.09 | -0.14 | 0.29 | -0.12 | 0.05 | -0.08 | -0.41 |  |  |  |
| (17) Govt Health Websites | 0.03 | 0.19 | -0.03 | 0.05 | 0.13 | -0.17 | 0.26 | -0.24 | -0.40 | -0.18 | 0.55 | -0.26 | 0.32 | 0.13 | 0.08 | 0.13 |  |  |
| (18) Training ICT health | 0.08 | -0.02 | -0.02 | 0.12 | -0.05 | 0.04 | 0.04 | 0.04 | -0.12 | -0.19 | 0.10 | -0.07 | 0.24 | 0.13 | 0.07 | -0.02 | 0.33 |  |
| (19) National EHR | 0.09 | -0.02 | 0.01 | 0.07 | 0.00 | -0.09 | 0.07 | -0.07 | -0.20 | -0.12 | -0.04 | 0.04 | 0.19 | 0.06 | 0.23 | -0.04 | 0.16 | 0.18 |
