# Supplementary material for: The Role of Information and Communications Technology Policies and Infrastructure in Curbing the Spread of the Novel Coronavirus: Cross-country Comparative Study
Source: JMIR Public Health Surveill. 2022 Jan 7;8(1):e31066. doi: 10.2196/31066 (PMC8745697; doi:10.2196/31066)
Supplement: Multimedia Appendix 2 [file publichealth_v8i1e31066_app2.docx]

**Multimedia Appendix 2.** Regression results for the sample with less population density (lower 70%).

**Table S1.** Regression results for the sample with less population density (lower 70%): log (total cases).

| Variables | DV^a^: log (Total Cases) | | | | | |
| --- | --- | --- | --- | --- | --- | --- |
|  |  | | | | | |
|  | (1)  Baseline Model | *P* value | (2)  Including Culture | *P* value | (3)  Interaction Effects | *P* value |
| log (GDP PPP) | 1.203 | <.001 | 1.435 | <0.001 | 1.416 | <0.001 |
|  | (0.154)^b^ |  | (0.15) |  | (0.162) |  |
| Unemployment Rate | 0.028 | 0.12 | 0.021 | 0.22 | 0.014 | 0.47 |
|  | (0.018) |  | (0.017) |  | (0.019) |  |
| log (Population Density) | 0.313 | 0.20 | -0.133 | 0.61 | -0.285 | 0.38 |
|  | (0.241) |  | (0.258) |  | (0.318) |  |
| Percent Ages 60 or Older | -0.021 | 0.22 | -0.012 | 0.57 | 0.005 | 0.85 |
|  | (0.017) |  | (0.021) |  | (0.025) |  |
| log (Annual Rainfall) | -0.067 | 0.81 | 0.245 | 0.50 | 0.175 | 0.65 |
|  | (0.278) |  | (0.355) |  | (0.381) |  |
| Annual Temperature | -0.02 | 0.13 | -0.02 | 0.21 | -0.015 | 0.41 |
|  | (0.013) |  | (0.016) |  | (0.017) |  |
| Early Lockdown | -0.119 | 0.52 | 0.061 | 0.72 | 0.041 | 0.82 |
|  | (0.184) |  | (0.166) |  | (0.183) |  |
| Individualism |  |  | -0.007 | 0.36 | -0.011 | 0.23 |
|  |  |  | (0.007) |  | (0.008) |  |
| Uncertainty Avoidance |  |  | -0.003 | 0.61 | -0.006 | 0.38 |
|  |  |  | (0.006) |  | (0.006) |  |
| **Distancing Enabling ICT Infrastructure** | | | | | | |
| Credit Card Ownership Rate | -0.006 | 0.35 | -0.004 | 0.46 | -0.007 | 0.31 |
|  | (0.007) |  | (0.006) |  | (0.006) |  |
| log (Broadband Speed) | 0.088 | 0.84 | 0.041 | 0.92 | 0.114 | 0.81 |
|  | (0.433) |  | (0.402) |  | (0.465) |  |
| **Medical ICT Infrastructure** | | | | | | |
| Telehealth Policy (Stated) | 0.004 | 0.98 | -0.133 | 0.50 | 0.153 | 0.80 |
|  | (0.208) |  | (0.193) |  | (0.588) |  |
| Telehealth Policy (Implied) | 0.006 | 0.98 | -0.274 | 0.13 | 0.309 | 0.49 |
|  | (0.186) |  | (0.178) |  | (0.44) |  |
| Govt Health Websites | -0.255 | 0.23 | -0.510 | 0.02 | -0.456 | 0.13 |
|  | (0.21) |  | (0.211) |  | (0.294) |  |
| Training ICT Health | 0.26 | 0.22 | 0.211 | 0.29 | 0.488 | 0.098 |
|  | (0.209) |  | (0.194) |  | (0.284) |  |
| National EHR | 0.061 | 0.72 | -0.104 | 0.50 | -0.163 | 0.503 |
|  | (0.167) |  | (0.152) |  | (0.24) |  |
| **Interaction** | | | | | | |
| Telehealth Policy (Stated)  *Govt Health Websites |  |  |  |  | -0.334 | 0.45 |
|  |  |  |  |  | (0.432) |  |
| Telehealth Policy (Implied)  *Govt Health Websites |  |  |  |  | -0.008 | 0.99 |
|  |  |  |  |  | (0.398) |  |
| Telehealth Policy (Stated)  *Training ICT Health |  |  |  |  | -0.341 | 0.62 |
|  |  |  |  |  | (0.685) |  |
| Telehealth Policy (Implied)  *Training ICT Health |  |  |  |  | -0.771 | 0.09 |
|  |  |  |  |  | (0.431) |  |
| Telehealth Policy (Stated)  *National EHR |  |  |  |  | 0.232 | 0.62 |
|  |  |  |  |  | (0.457) |  |
| Telehealth Policy (Implied)  *National EHR |  |  |  |  | 0.089 | 0.82 |
|  |  |  |  |  | (0.396) |  |
| Constant | -9.420 | <0.001 | -11.188 | <0.001 | -10.822 | <0.001 |
|  | (1.772) |  | (1.823) |  | (1.97) |  |
| Observations, n | 73 | | 47 | | 47 | |
| R^2^ | 0.612 | | 0.835 | | 0.859 | |
| Adjusted R^2^ | 0.518 | | 0.747 | | 0.729 | |

^a^ DV: Dependent Variable

^b^ Standard errors in parentheses

^c^ N/A: Not applicable

**Table S2.** Regression results for the sample with less population density (lower 70%): fatality rate.

| Variables | DV^a^: Fatality Rate | | | | | |
| --- | --- | --- | --- | --- | --- | --- |
|  |  | | | | | |
|  | (1)  Baseline Model | *P* value | (2)  Including Culture | *P* value | (3)  Interaction Effects | *P* value |
| log (GDP PPP) | 0.746 | 0.19 | 0.957 | 0.28 | 0.982 | 0.32 |
|  | (0.561)^b^ |  | (0.873) |  | (0.97) |  |
| Unemployment Rate | -0.065 | 0.31 | -0.07 | 0.48 | -0.063 | 0.58 |
|  | (0.064) |  | (0.098) |  | (0.113) |  |
| log (Population Density) | 0.08 | 0.93 | 0.838 | 0.58 | 1.307 | 0.50 |
|  | (0.909) |  | (1.498) |  | (1.897) |  |
| Percent Ages 60 or Older | 0.058 | 0.35 | 0.051 | 0.68 | 0.042 | 0.78 |
|  | (0.061) |  | (0.123) |  | (0.149) |  |
| log (Annual Rainfall) | -1.01 | 0.33 | 0.647 | 0.76 | 0.402 | 0.86 |
|  | (1.033) |  | (2.062) |  | (2.276) |  |
| Annual Temperature | 0.012 | 0.81 | 0.007 | 0.94 | 0.005 | 0.96 |
|  | (0.05) |  | (0.091) |  | (0.103) |  |
| Early Lockdown | 0.246 | 0.72 | -0.208 | 0.83 | 0.143 | 0.90 |
|  | (0.682) |  | (0.961) |  | (1.095) |  |
| Individualism |  |  | 0.065 | 0.14 | 0.07 | 0.75 |
|  |  |  | (0.042) |  | (0.05) |  |
| Uncertainty Avoidance |  |  | 0.023 | 0.50 | 0.03 | 0.18 |
|  |  |  | (0.034) |  | (0.038) |  |
| **Distancing Enabling ICT Infrastructure** | | | | | | |
| Credit Card Ownership Rate | 0.015 | 0.53 | -0.01 | 0.76 | -0.012 | 0.44 |
|  | (0.024) |  | (0.033) |  | (0.038) |  |
| log (Broadband Speed) | 0.555 | 0.72 | -0.29 | 0.90 | 0.535 | 0.85 |
|  | (1.555) |  | (2.336) |  | (2.779) |  |
| **Medical ICT Infrastructure** | | | | | | |
| Telehealth Policy (Stated) | -1.393 | 0.07 | -1.903 | 0.10 | -1.19 | 0.74 |
|  | (0.763) |  | (1.121) |  | (3.51) |  |
| Telehealth Policy (Implied) | -1.636 | 0.02 | -2.392 | 0.03 | -3.829 | 0.16 |
|  | (0.673) |  | (1.033) |  | (2.625) |  |
| Govt Health Websites | -0.091 | 0.91 | -1.043 | 0.40 | -0.795 | 0.66 |
|  | (0.771) |  | (1.222) |  | (1.755) |  |
| Training ICT Health | -0.477 | 0.54 | -0.515 | 0.65 | -0.733 | 0.67 |
|  | (0.779) |  | (1.127) |  | (1.694) |  |
| National EHR | -0.598 | 0.33 | 0.407 | 0.65 | -0.616 | 0.67 |
|  | (0.602) |  | (0.882) |  | (1.432) |  |
| **Interaction** | | | | | | |
| Telehealth Policy (Stated)  *Govt Health Websites |  |  |  |  | -0.173 | 0.95 |
|  |  |  |  |  | (2.577) |  |
| Telehealth Policy (Implied)  *Govt Health Websites |  |  |  |  | -1.475 | 0.54 |
|  |  |  |  |  | (2.375) |  |
| Telehealth Policy (Stated)  *Training ICT Health |  |  |  |  | -1.387 | 0.74 |
|  |  |  |  |  | (4.09) |  |
| Telehealth Policy (Implied)  *Training ICT Health |  |  |  |  | 1.857 | 0.48 |
|  |  |  |  |  | (2.571) |  |
| Telehealth Policy (Stated)  *National EHR |  |  |  |  | 1.424 | 0.61 |
|  |  |  |  |  | (2.729) |  |
| Telehealth Policy (Implied)  *National EHR |  |  |  |  | 2.542 | 0.29 |
|  |  |  |  |  | (2.366) |  |
| Constant | -3.888 | 0.56 | -12.013 | 0.27 | -14.288 | 0.24 |
|  | (6.663) |  | (10.58) |  | (11.761) |  |
| Observations, n | 71 | | 47 | | 47 | |
| R^2^ | 0.291 | | 0.378 | | 0.436 | |
| Adjusted R^2^ | 0.113 | | 0.046 | | -0.081 | |

^a^ DV: Dependent Variable

^b^ Standard errors in parentheses

^c^ N/A: Not applicable

**Table S3.** Regression results for the sample with less population density (lower 70%): log (number of days).

| Variables | DV^a^: log (Number of Days) | | | | | |
| --- | --- | --- | --- | --- | --- | --- |
|  |  | | | | | |
|  | (1)  Baseline Model | *P* value | (2)  Including Culture | *P* value | (3)  Interaction Effects | *P* value |
| log (GDP PPP) | -0.154 | 0.007 | -0.135 | 0.08 | -0.125 | 0.096 |
|  | (0.055)^b^ |  | (0.075) |  | (0.072) |  |
| Unemployment Rate | 0.002 | 0.77 | -0.0002 | 0.99 | 0.005 | 0.53 |
|  | (0.006) |  | (0.008) |  | (0.008) |  |
| log (Population Density) | -0.186 | 0.04 | -0.276 | 0.04 | -0.163 | 0.26 |
|  | (0.088) |  | (0.128) |  | (0.141) |  |
| Percent Ages 60 or Older | 0.003 | 0.65 | 0.002 | 0.86 | -0.009 | 0.45 |
|  | (0.006) |  | (0.011) |  | (0.011) |  |
| log (Annual Rainfall) | 0.044 | 0.67 | -0.112 | 0.53 | -0.137 | 0.43 |
|  | (0.1) |  | (0.176) |  | (0.169) |  |
| Annual Temperature | 0.011 | 0.03 | 0.008 | 0.34 | 0.003 | 0.71 |
|  | (0.005) |  | (0.008) |  | (0.008) |  |
| Early Lockdown | -0.084 | 0.21 | -0.103 | 0.22 | -0.125 | 0.14 |
|  | (0.066) |  | (0.082) |  | (0.082) |  |
| Individualism |  |  | -0.003 | 0.44 | -0.002 | 0.62 |
|  |  |  | (0.004) |  | (0.004) |  |
| Uncertainty Avoidance |  |  | -0.002 | 0.57 | -0.001 | 0.78 |
|  |  |  | (0.003) |  | (0.003) |  |
| **Distancing Enabling ICT Infrastructure** | | | | | | |
| Credit Card Ownership Rate | -0.001 | 0.71 | -0.002 | 0.39 | 0.0002 | 0.94 |
|  | (0.002) |  | (0.003) |  | (0.003) |  |
| log (Broadband Speed) | 0.007 | 0.96 | -0.0004 | 0.998 | -0.111 | 0.60 |
|  | (0.151) |  | (0.2) |  | (0.207) |  |
| **Medical ICT Infrastructure** | | | | | | |
| Telehealth Policy (Stated) | -0.035 | 0.64 | -0.023 | 0.81 | -0.266 | 0.32 |
|  | (0.074) |  | (0.096) |  | (0.261) |  |
| Telehealth Policy (Implied) | -0.019 | 0.78 | 0.044 | 0.63 | -0.387 | 0.06 |
|  | (0.065) |  | (0.088) |  | (0.195) |  |
| Govt Health Websites | 0.073 | 0.33 | 0.154 | 0.15 | 0.155 | 0.25 |
|  | (0.075) |  | (0.105) |  | (0.131) |  |
| Training ICT Health | 0.004 | 0.96 | -0.015 | 0.88 | -0.269 | 0.04 |
|  | (0.076) |  | (0.096) |  | (0.126) |  |
| National EHR | -0.035 | 0.56 | -0.009 | 0.90 | 0.05 | 0.65 |
|  | (0.059) |  | (0.075) |  | (0.107) |  |
| **Interaction** | | | | | | |
| Telehealth Policy (Stated)  *Govt Health Websites |  |  |  |  | -0.087 | 0.65 |
|  |  |  |  |  | (0.192) |  |
| Telehealth Policy (Implied)  *Govt Health Websites |  |  |  |  | 0.113 | 0.53 |
|  |  |  |  |  | (0.177) |  |
| Telehealth Policy (Stated)  *Training ICT Health |  |  |  |  | 0.394 | 0.21 |
|  |  |  |  |  | (0.305) |  |
| Telehealth Policy (Implied)  *Training ICT Health |  |  |  |  | 0.541 | 0.009 |
|  |  |  |  |  | (0.191) |  |
| Telehealth Policy (Stated)  *National EHR |  |  |  |  | -0.001 | 0.996 |
|  |  |  |  |  | (0.203) |  |
| Telehealth Policy (Implied)  *National EHR |  |  |  |  | -0.198 | 0.27 |
|  |  |  |  |  | (0.176) |  |
| Constant | 3.422 | <0.001 | 3.956 | <0.001 | 4.097 | <0.001 |
|  | (0.648) |  | (0.905) |  | (0.876) |  |
| Observations, n | 71 | | 47 | | 47 | |
| R^2^ | 0.394 | | 0.464 | | 0.632 | |
| Adjusted R^2^ | 0.242 | | 0.179 | | 0.295 | |

^a^ DV: Dependent Variable

^b^ Standard errors in parentheses

^c^ N/A: Not applicable
